# Changes in the Physiological and Morphometric Characteristics and Biomass Distribution of Forage Grasses Growing under Conditions of Drought and Silicon Application

**DOI:** 10.3390/plants12010016

**Published:** 2022-12-20

**Authors:** Grażyna Mastalerczuk, Barbara Borawska-Jarmułowicz, Ahmad Darkalt

**Affiliations:** 1 Department of Agronomy, Institute of Agriculture, Warsaw University of Life Sciences—SGGW, Nowoursynowska 159 St., 02-776 Warsaw, Poland; 2 Department of Renewable Natural Resources & Ecology, Engineering Agricultural Faculty, Aleppo University, Aleppo 12212, Syria

**Keywords:** biomass distribution, C:N ratio, drought, *Festuca arundinacea*, *Festulolium braunii*, gas exchange parameters, *Lolium perenne*, roots, silicon fertilization

## Abstract

Research on mitigating the effects of water scarcity by applying silicon to perennial grasses is still insufficient. This study was conducted to investigate the effect of spring and summer droughts and silicon applications on gas exchange parameters; the morphometric characteristics of root systems; and the biomass distribution of *Festulolium braunii*, *Festuca arundinacea*, and *Lolium perenne* cultivars. Plants were treated with a drought during the tillering phase once a year (during spring or summer regrowth) for 21 days. Foliar nutrition with silicon was applied twice under the drought conditions. Grasses in a pot experiment were cut three times during vegetation. The plants that were exposed to the drought had lower values of the gas exchange parameters than those that were well watered. The beneficial effect of silicon was related to the reduction of excessive water loss through transpiration during the spring drought. Under the drought and silicon applications, the water use efficiency, root dry mass, and length increased compared to the control. Moreover, silicon increased the proportion of both the finer and thicker roots in *F. braunii* and *L. perenne*, while the distribution of the root diameter changed least in the more resistant *F. arundinacea*. Silicon also reduced the carbon content in the roots and increased root carbon accumulation. Our results indicated that Si may help perennial forage grasses cope better with drought stress. This was due to the allocation of carbon to the roots to develop the fine root network, increasing the length and root biomass and improving the water use efficiency.

## 1. Introduction

Water deficits are one of the most serious environmental stresses that have been repeated in recent years in Central Europe, including in Poland. Extreme weather events, such as severe droughts and heat waves, are also expected to increase in magnitude and frequency in the near future [1]. Droughts are one of the most important abiotic stressors affecting the growth and productivity of agricultural crops. They can occur with varying intensity, duration, and also at different times of the year [2]. Spring droughts in grasslands usually reduce the yield of the first growth of meadow plants and the productivity of pastures, while summer droughts affect the second growth of swards [3]. Frequent precipitation deficits not only reduce grassland productivity but also lead to negative changes in the ecosystem structure and the carbon balance [4]. 

To grow and develop properly, plants take up water from substrate and assimilated atmospheric CO_2_, which they then use in photosynthesis. These activities are possible thanks to transpiration. This process is mediated by the stomata and plays a key role in gas exchange, water uptake, and thermoregulation [5]. Since water and carbon dioxide are substrates in photosynthesis, transpiration has a direct impact on the biomass increase and, thus, on plant productivity. Droughts adversely affect the physiological processes in plants and interfere with the uptake and transport of nutrients. Photosynthesis is a key process for grassland growth and yield and is directly affected by the leaf water content [6]. Under the condition of a water deficit, the plant water potential, relative leaf water content, and photosynthetic rate decrease. Plants reduce water loss by closing their stomata. Under such conditions, less diffusion of CO_2_ from the atmosphere to the carboxylation sites in leaves is observed [7]. Drought stress leads to the loss of photosynthetic pigments, impairs enzyme activity, and also impairs photosystem II activity [8]. Plant susceptibility to water deficit stress varies and depends on the plant species and its growth stage as well as the stress duration, severity, and other various accompanying stress factors [9].

Grass species from the genera *Lolium* and *Festuca* are the main forage grasses grown in the temperate climate zone. They are characterized by different forage qualities and tolerances to changes in soil water conditions. *Lolium perenne* is a highly stocky grass that provides high grass yields with a good forage value for ruminants. It is better adapted to grazing than other temperate grass species. However, this species requires a relatively large amount of water to maintain its growth. *L. perenne* is also known to respond quickly to water shortages. It belongs to shallow-rooting grasses with a limited drought tolerance [10]. The forage quality of *Festuca arundinacea* is not as good as that of *L. perenne*, even under its optimal soil and aerial conditions. Nevertheless, it has a great potential for developing a deep and extensive root system and is able to tolerate water deficits by reprogramming the cellular metabolism in its leaves and other organs [11]. *Festulolium braunii*, in turn, a generic hybrid of the *Festuca* and *Lolium* genera, is characterized by a high yield potential and a forage quality similar to the *Lolium* species as well as persistence and stress tolerance comparable to *Festuca* [12,13]. 

As a result of the progressive climate change, more attention is being paid to the possibility of using different growth stimulants to obtain high quality plants [14]. In the case of grasslands, the solution to this problem may lie in exploring the effects of using fertilizers containing silicon (Si) on the yield and forage value of swards [15,16]. Si plays an essential role in the plant cycle as a macroelement. This element is the eighth most abundant element in nature and the second most abundant element in the soil after oxygen. In the soil solution, Si occurs mainly as monosilicic acid (H_4_SiO_4_) and is taken up by plants in this form. Si is beneficial for the growth, development, and yield of many plant species [17]. The positive effects of Si applications have been reported, including improvements in the intensity of the physiological and metabolic processes [18,19], an increased insect and disease resistance [20], an improvement in the nutrient imbalance [21], and an improved tolerance to salt [22] and droughts [23,24]. In response to droughts, plants stimulate the production of reactive oxygen species (ROS), which causes membrane injuries, protein degradation, and enzyme inactivation, therefore triggering oxidative stress [25]. The application of Si can improve the activity of antioxidant enzymes and the concentration of antioxidant metabolites in plants growing under water stress [26]. In addition, Si has a positive effect on root system development [27]. Plants respond differently to Si fertilization, and their ability to take up Si from the soil solution differs [21]. Therefore, it is necessary to continue experiments that will allow us to better understand the interactions between Si fertilization and plant responses, especially for grass species under drought conditions.

Many different drought-related characteristics of plants are being investigated in studies, but they are only being conducted on above- or below-ground plant parts. This makes studies of grass tolerance to water deficits difficult to compare. Detailed information on the simultaneous effects of droughts on the photosynthetic performance, biomass distribution, and root morphometric characteristics of species of perennial forage grasses is lacking.

Given the scarcity of freshwater resources, it is not only important to search for grass species with a good drought tolerance but also for methods to increase plant resistance to this stress. The objective of this work was to investigate the effects of water deficits and Si use on photosynthesis and how the latter could alter the root system of forage grasses. Therefore, we investigated the changes in the gas exchange parameters, physiological characteristics, root system morphological traits and biomass distribution of *Festulolium braunii*, *Festuca arundinacea*, and *Lolium perenne* cultivars in response to Si applications under drought conditions. The subjects of the study were the mature plants of perennial forage grasses in the tillering stage that were exposed to drought stress during the period when the plants were most exposed to water deficits under natural conditions, i.e., spring and summer.

## 2. Results

**Figure 1:**
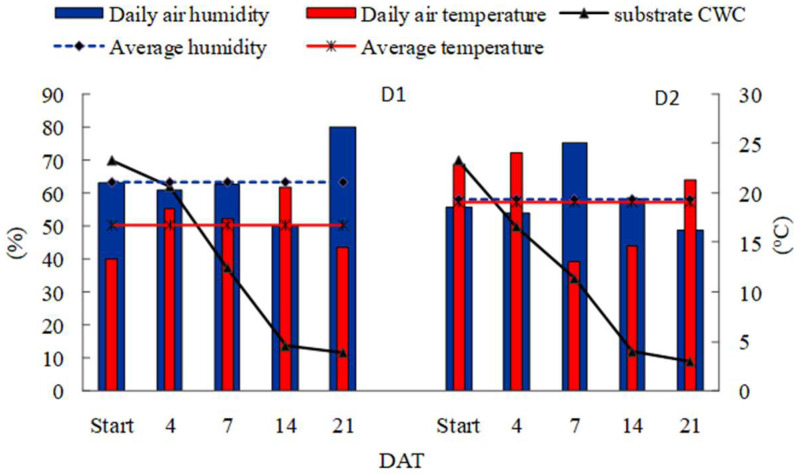
Average daily air humidity (%), air temperature (°C), and substrate capillary water capacity—CWC (%)—in the following days of water deficit treatment (4, 7, 14, and 21 DAT) during spring and summer droughts (D1 and D2, respectively).

The capillary water capacity (CWC) of the substrate decreased from 70% to approximately 10% under drought conditions (D1, D2) (Figure 1). The spring drought (D1) was characterized by moderate humidity (approx. 64%) and the daily air temperature (16.8 °C). During the summer drought (D2), a higher air temperature and lower humidity (19.2 °C and 58%, respectively) than those in D1 were recorded, which, under the conditions of the experiment, could intensify the effect of the drought.

**Figure 2:**
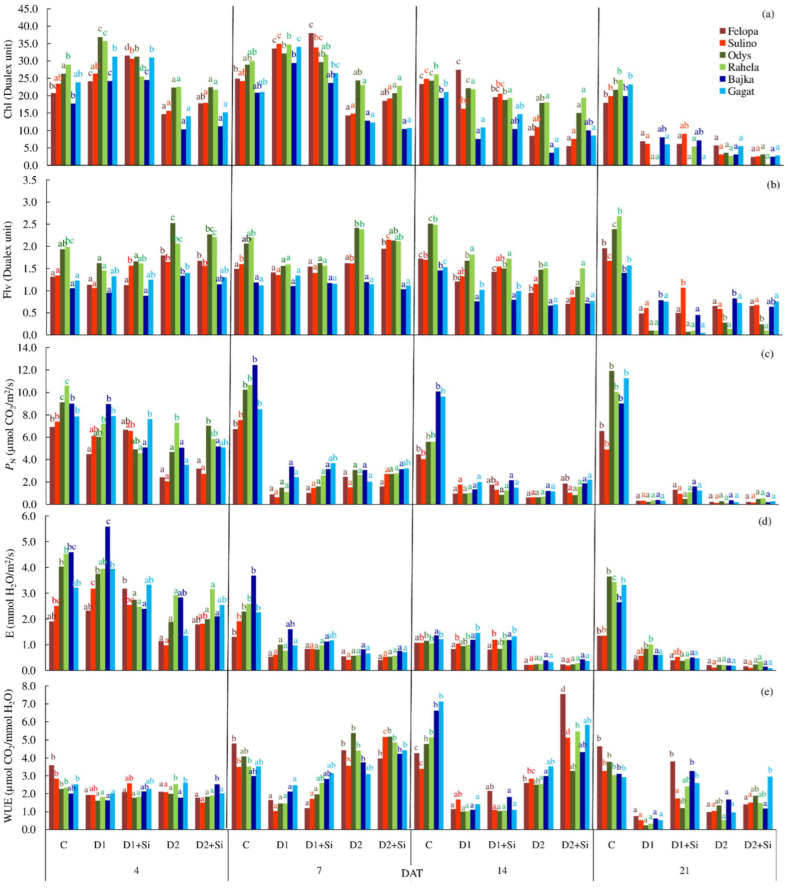
Variations in (**a**) content of chlorophyll (Chl), (**b**) content of flavonols (Flv), (**c**) net photosynthetic rate (*P*_N_), (**d**) transpiration rate (*E*), and (**e**) water use efficiency (WUE) between cultivars of *F. braunii* (cvs. of Felopa and Sulino), *F. arundinacea* (cvs. of Odys and Rahela), and *L. perenne* (cvs. of Bajka and Gagat) in terms of measurement (4, 7, 14, or 21 days after treatment) depending on water conditions and terms of silicon application (C—control, D1—spring drought, D1 + Si—spring drought with Si application, D2—summer drought, and D2 + Si—summer drought with Si application); the means (bars with the same color) marked with the same lower-case letters (the same color) do not differ significantly according to Tukey’s test (*p* ≤ 0.05).

There were significant differences in the physiological features of the plants under different water conditions and Si applications (Table 1). In general, the drought conditions differentiated the average values of Chl and Flv, decreasing them relative to the control, independent of the Si application and the term of drought (D1, D2). However, the plant response changed with the duration of the drought (Figure 2a). An increase in the values of Chl compared to the control were observed in D1 and D1 + Si, especially in the first week of stress, whereas the plants were characterized by similar or lower Chl contents in D2 and D2 + Si. In the case of the Flv content, higher values in relation to the control were found in the first days of stress only in the plants in the summer drought (Figure 2b). There were also differences in the values of Chl and Flv between the species and cultivars (Table 1 and Table 2). *F. arundinacea* cvs. showed the highest values of the features, while *L. perenne* cvs. showed the lowest values of features.

Plants exposed to drought conditions showed noticeably lower average values of the net photosynthetic (*P*_N_) and transpiration rates (*E*) as well as the stomatal conductance (*g*_s_) than those of plants under optimal substrate moisture conditions (C) (Table 1). At the same time, the *E* and *g*_s_ were higher during D1 than during D2. It was shown that the responses of plants to the droughts in the following days varied. Relatively high values of *P*_N_ and *E* were recorded only during the fourth day after treatment. At the subsequent terms of measurement (except 14 DAT in relation to *E*), the values of these parameters in the conditions of water deficiency and Si fertilization were significantly lower compared to the control (Figure 2c,d). The results also showed that the cultivars of *F. braunii* were characterized by the lowest values of the gas exchange parameters (*P*_N_, *E*, *g*_s_, *C*_i_) regardless of other factors. There was only a slight difference in the water use efficiency (WUE) between the tested species. However, the drought conditions resulted in the less effective water management of plants. The effect of Si was evident, especially during the 14th DAT of D2 and the 21st DAT in the case of D1 (Figure 2e). The results also demonstrated a significant reduction in the relative water content of the leaves (RWC) under each drought. Simultaneously, the application of Si significantly increased the RWC values under the D1 condition. Moreover, the RWC varied in the tested species. The highest values were obtained from the *F. braunii* cvs., while the lowest were obtained from *F. arundinacea*.

The biometric parameters of the plant roots changed depending on the water conditions, Si applications, tested species, and cultivars (Table 1). The spring drought (D1) only slightly reduced the average diameter of the plant roots (RD). The influence of the species and cultivars on the RD values was noted as well. The cultivars of *F. arundinacea*, especially Odys, were characterized by the highest values of the RD. The length of the roots (RL) was affected by the droughts and Si applications. The spring drought reduced the RL by approx. 50% compared to the control conditions, while the summer drought reduced the RL by approx. 40%. Simultaneously, the application of Si, in both cases, significantly increased the length of the roots (approx. 15%). The values of the RL parameter were different between the tested species and cultivars. The highest values were found in the cvs. of *L. perenne*, whereas the lowest values were found in the cvs. of *F. arundinaceae*.

**Figure 3:**
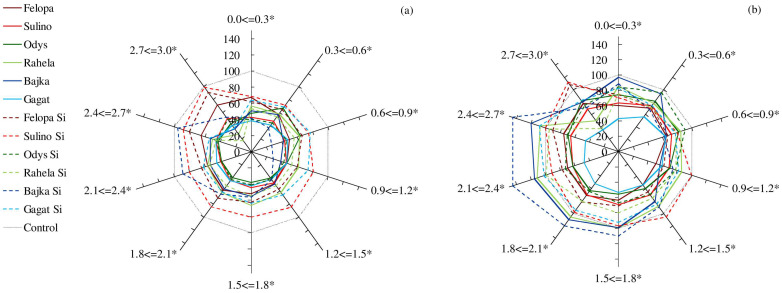
Root length distribution (% relative to control) of *F. braunii* (cvs. of Felopa and Sulino), *F. arundinacea* (cvs. of Odys and Rahela), and *L. perenne* (cvs. of Bajka and Gagat) in ten diameter classes (0.0 <= 0.3, 0.3 <= 0.6, 0.6 <= 0.9, 0.9 <= 1.2, 1.2 <= 1.5, 1.5 <= 1.8, 1.8 <= 2.1, 2.1 <= 2.4, 2.4 <= 2.7, and 2.7 <= 3 mm) depending on the term of water deficit, (**a**) spring drought (D1) and (**b**) summer drought (D2); *—significant differences in interaction cultivars and water and Si conditions (*p* ≤ 0.05).

The research also showed the effect of the water shortages and Si applications on the distribution of the root lengths in the individual diameter classes (Figure 3). 

The spring drought (D1) caused a strong reduction in the length of the roots in all the defined diameter classes in relation to the control in all the species and cultivars. Simultaneously, under the drought conditions and Si applications, both the cvs. of *F. braunii* and the cv. of Bajka (*L. perenne*) were distinguished by having thicker roots than they did in the case of a drought without fertilization. The summer drought (D2) diversified the distribution of the root lengths of the plants in the individual diameter classes differently than the spring drought did. The Bajka cv. was characterized by the highest share of thicker roots with a diameter of 1.5–2.7 mm. The application of Si modified the distribution of the *F. arundinacea* roots the least, especially in the Rahela cv. In the case of the other species and cultivars (expect the Bajka cv.), Si fertilization before D2 increased the lengths of the roots in all the defined classes of diameter. 

The drought stress significantly differentiated the mass of the plants compared with the control conditions (Table 1). The values of total plant dry mass (TPDM), shoot dry mass (SDM), and root dry mass (RDM) decreased the most when plants were subjected to a drought during spring regrowth. In the second regrowth, the reduction in the plant biomass due to the drought was smaller. A significant increase in the RDM and TPDM were demonstrated as a result of the Si applications. In the case of the SDM, fertilization with Si caused an increase in the obtained values in D2 only. The cultivars of Rahela and Odys (*F. arundinacea*) were distinguished by the highest values of the RDM and TPDM, whereas the Bajka cv. (*L. perenne*) was characterized by a significantly lower SDM and RDM compared to those of others. The parameters reflecting the root fineness (SRL) and that were related to the allocation of biomass to the roots (RMR) also varied depending on the water conditions and fertilization with Si. The highest SRL values were found in the plants subjected to the droughts, especially in the spring (D1). Meanwhile, the highest values of the RMR under Si application before the spring drought were noted. The cultivars of *L. perenne* were found to have the finest roots. Simultaneously, both the cultivars of *F. arundinacea* showed the highest RMR and the lowest SRL values relative to those of the others.

**Figure 4:**
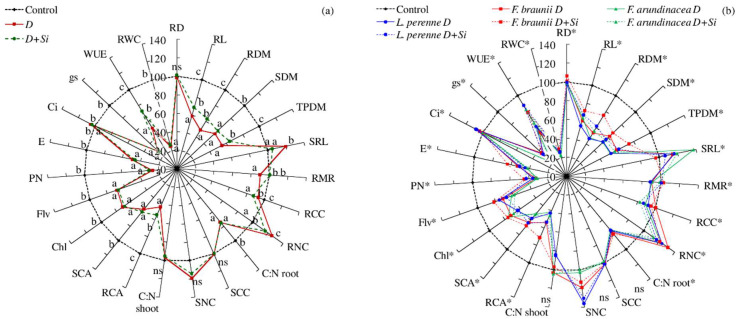
Variability of the values of the examined features depending on the drought (D) and application of silicon before drought (D + Si) (% in relation to control—C); (**a**) mean values of features irrespective of the species and cultivars and (**b**) mean values of features for the tested species (*F. braunii*, *F. arundinacea*, *L. perenne*) under the water and Si conditions (C, D, and D + Si); Chl—content of chlorophyll, *C*_i_—sub-stomatal CO_2_ concentration, *E*—transpiration rate, Flv—content of flavonols, *g*_s_—stomatal conductance, *P*_N_—net photosynthetic rate, RCA—root carbon accumulation, RCC—root carbon content, RD—root diameter, RDM—root dry mass, RL—total root length, RMR—root mass ratio, RNC—root nitrogen content, root C:N—root carbon-to-nitrogen ratio, RWC—relative water content, SCA—shoot carbon accumulation, SCC—shoot carbon content, SDM—shoot dry mass, shoot C:N—shoot carbon-to-nitrogen ratio, SNC—shoot nitrogen content, SRL—specific root length, TPDM—total plant dry mass, WUE—water use efficiency; different lowercase letters indicate significant differences among water and Si conditions (C, D, and D + Si) (*p* ≤ 0.05), ns—not significant, and *—significant differences for interaction species and water and Si conditions (*p* ≤ 0.05).

The average content of carbon in the plant shoots (SCC), regardless of the species or cultivar, was approx. 13% higher compared to the content in the roots (RCC). The species and cultivars as well as the droughts and Si applications had no effect on the SCC, but they differentiated the RCC (Table 1). Both the cultivars of *F. arundinacea* were characterized by the highest RCCs. The droughts limited the RCC, especially when the plants were fertilized with Si. The carbon accumulation in the roots (RCA) and shoots (SCA) of the plants changed depending on the droughts and Si applications. Fertilization with Si before D1 and D2 significantly increased RCA as compared to the control, while the SCA values were higher only in the case of applying Si before D2. This study showed that the cvs. of *F. arundinaceae* showed the highest values of RCA and were distinguished by the lowest content of nitrogen in their shoots (SNC) and roots (RNC). Both the droughts and the applications of Si increased the RNC. The effect of water deficits and Si fertilization on the SNC was varied. D1 and D1 + Si applied during the first regrowth increased the SNC, while the plants treated with D2 and D2 + Si during the second regrowth showed a reduction in the values of this parameter. A significant decrease in the root C:N ratio was shown as a result of the droughts. In the case of the shoots, a significant decrease in the C:N ratio during the spring drought and a significant increase during the summer drought in relation to the control were observed. The values of the C:N ratio in the shoots and roots were significantly higher in the cultivars of *F. arundinacea*. Regardless of the cultivar of the grasses or the term of the stress, it was found that the water conditions and the application of Si caused changes in the values of most of the tested parameters. Only the RD, SCC, SNC, and also C:N ratio in the shoots remained unchanged in relation to the control conditions (Figure 4a). 

**Figure 5:**
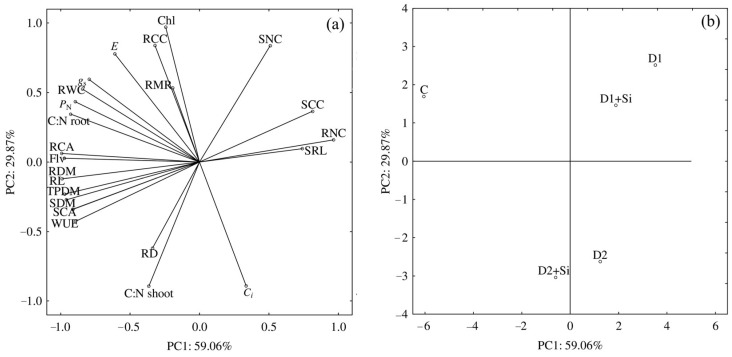
Results of principal component analysis (PCA) (**a**) presenting relationships between all analyzed parameters and (**b**) for the conditions of water and Si application (C—control, D1—spring drought, D1 + Si—spring drought with Si application, D2—summer drought, D2 + Si—summer drought with Si application); Chl—content of chlorophyll, *C_i_*—sub-stomatal CO_2_ concentration, *E*—transpiration rate, Flv—content of flavonols, *g_s_*—stomatal conductance, *P*_N_—net photosynthetic rate, RCA—root carbon accumulation, RCC—root carbon content, RD—root diameter, RDM—root dry mass, RL—total root length, RMR—root mass ratio, RNC—root nitrogen content, root C:N—root carbon-to-nitrogen ratio, RWC—relative water content, SCA—shoot carbon accumulation, SCC—shoot carbon content, SDM—shoot dry mass, shoot C:N—shoot carbon-to-nitrogen ratio, SNC—shoot nitrogen content, SRL—specific root length, TPDM—total plant dry mass, and WUE—water use efficiency.

PCA analysis showed that the first component accounted for 59.06% of the analyzed variability, and the second accounted for 29.87% of the analyzed variability (Figure 5a,b). The water conditions and Si applications had the highest impact on the values of the RCA, Flv, RDM and RL and had a slightly less, but significant, impact on the TPDM, SDM, SCA and WUE. These parameters were significantly and positively correlated with each other and were negatively correlated with the RNC, SRL and SCC. 

Moreover, the analysis showed that the parameters of the RWC, *P*_N_, *g*_s_, and root C:N ratio were strongly and positively correlated with each other. The application of Si under the droughts significantly increased the RL, RDM, TPDM, RCA, RMR, and WUE (Figure 4a). Simultaneously, the plants of all the species that were fertilized with Si before the drought were characterized by lower values of the SRL, RNC, and RCC than those of the plants that were subjected to the droughts without Si (Figure 4b).

## 3. Discussion

The drought applied in our study had a detrimental effect on the plants, but the effect was variable and depended on the duration of the droughts and Si applications as well as on the species and varieties studied.

The presence of chlorophyll in the leaves is essential to the process of photosynthesis, as it helps harvest light energy to drive the electron transport reactions [28]. The differentiation of the chlorophyll content in plants depends on the species and cultivars, plant developmental stage, nutrient availability, and weather and soil conditions [29]. Our studies revealed that both the species and drought conditions differentiated the Chl content, and the level of SPAD units increased the most in D1 in the first seven DAT compared to the control. At the same time, the prolongation of the stress showed a significant decrease in the chlorophyll content of the plants. In our experiment, the *F. arundinacea* cultivars showed the highest levels of Chl, and the *L. perenne* cultivars showed the lowest levels of Chl. The literature data and our previous studies confirmed that stress sensitivity can be correlated with the extent of chlorophyll degradation induced by a moisture deficit [6,30]. According to Li et al. [31], the components of the photosynthetic apparatus could be damaged in drought-sensitive genotypes, whereas drought-tolerant genotypes have a relatively good adaptability for reducing or avoiding the impairment caused by drought stress. One of the basic mechanisms of plant drought acclimation is the accumulation of non-enzymatic flavonoids, which increase the antioxidant capacity and play a role in detoxifying the reactive oxygen species formed under drought stress. They are localized in the epidermal cells of the leaves and play an important role in protecting the photosynthetic apparatus [32]. Ma et al. [33] reported an increase in the total amount of flavonoids in the tolerant genotypes of wheat under drought conditions. In our study, the values of the Flv content were higher in the plants that suffered from the summer drought compared to the control. Among the tested plants, the more resistant cvs. of *F. arundinacea* were characterized by higher levels of these compounds under all the water and Si conditions, whereas Si supplementation in the drought conditions had the strongest effect on the susceptible cvs. of *F. braunii*, which reacted with an increase in the Flv content. The elevated levels of the flavonoid contents indicated that Si may be important in the antioxidant defenses that occurred in response to the droughts, especially in the more drought-sensitive grass species. The exogenous application of silicon could alleviate the oxidative stress of the plants through the modification of the biosynthesis of reactive species and the transcriptional regulation of multiple defense pathways, such as antioxidant enzymes, antioxidant active substances, and also flavonoid compounds [34,35].

Our studies showed that drought stress reduced the *g_s_*, *E*, and *P*_N_. At the phenotypic level, these responses manifested as reductions in the SDM. The observed changes were caused by stomata closure, a well-known response to droughts [36]. However, the stomata closure did not contribute to the decrease in CO_2_ in the intercellular spaces. This phenomenon can be partially explained by a decrease in the photosynthetic rate (reduction in CO_2_ consumption) and an increase in the metabolic CO_2_ production during stress. Our observations suggested that plants reduced water loss by limiting transpiration and attempted to cope with reduced energy production (the decrease in photosynthetic intensity) by increasing their catabolic processes and redistributing carbon [37]. Osmotic adjustment is an important mechanism of drought tolerance that helps retain water in plant tissues despite a low water potential [38]. 

In our studies, the use of Si under drought conditions did not significantly differentiate the values of most of the gas exchange parameters. The beneficial effect of Si was related to the reduction of excessive water loss through transpiration only under the spring drought conditions. According to Sacała [19], the beneficial effect of the Si fertilization of plants may be due to more effective osmoregulation, reduced water loss through transpiration, an improved water balance, the maintenance of an adequate supply of necessary nutrients, a reduced uptake of toxic ions, and also an improved performance of the antioxidant mechanisms.

Farooq et al. [39] indicated that drought tolerant species maintain their WUE by reducing water loss. However, in cases where plant growth is impeded to a greater extent, the WUE is also significantly reduced. In the studies by Hattori et al. [40,41], sorghum plants grown under a drought and in the presence of Si had a higher RWC and dry matter due to an improved shoot water uptake. Gong et al. [42] showed a similar dependence in wheat plants. Agarie et al. [43] found that the incorporation of Si into the plant cell wall reduced transpiration and increased the internal water storage under drought stress. In our study, the droughts reduced both the WUE—a measure of the amount of water transpired per given unit of CO_2_—and the RWC (also referred to as relative turgidity)—a measure of the plant water status that reflects the metabolic activity in the tissues and is also used as an important indicator of dehydration tolerance. At the same time, the application of Si during the period of intensive plant growth under the spring drought conditions (D1 + Si) increased the WUE and RWC values by 53.3% and 9.2%, respectively, while, under the summer drought conditions (D2 + Si), it increased by 31.4% and 3.9%, respectively (Table 1). The effect of Si on the WUE was particularly evident in most of the tested cultivars (with the exception of Odys) during the last stress period (14 and 21 DAT). 

According to Farooq et al. [39], the application of Si plays an important role in both water transport and the maintenance of root growth under drought stress. In the studies of Hattori et al. [41], Si also decreased the shoot-to-root ratio, indicating the facilitation of root growth and the maintenance of a higher photosynthetic rate and stomatal conductance compared to those of the plants grown without silicon application under a drought. In this study, we found that, under the droughts and Si applications, not only the WUE values but also the RL, RDM, and RMR of the plants increased significantly compared to the treatments without silicon (Figure 4). These results indicated that under the condition of Si fertilization, the tested grasses adapted to drought stress by developing roots that enabled their growth and reproduction and, accordingly, reducing the allocation of photosynthetic products to the aboveground organs (stems and leaves), which supported RCA. The RMR increased significantly under the D + Si treatment, especially in the spring. Hattori et al. [40] argued that the effect of Si stimulation on root growth could be due to enhanced root elongation caused by an increase in the cell wall extensibility in the growth region as observed in sorghum. At the same time, the current study showed that plants responded to D1 and D2 by distributing the carbon flowing from the leaf into the root system but that they used it primarily to form fine roots (with small diameters). As a result, the SRL of the *L. perenne* and *F. braunii* cvs. increased under the D1 and D2 treatments, while the RMR decreased. A likely explanation is that the sensitivity of the fine roots to moisture and nutrient availability was greater than that of the thick roots. The main physiological function of the fine roots is the uptake of water and nutrients, while the main function of the thick roots is support and transport [44,45]. The Si applications increased the proportion of both the finer and thicker roots in *F. braunii* and *L. perenne*. However, their SRL varied slightly and was more depended on the water deficit. The distribution of the root diameter in the defined length classes changed least in the more resistant *F. arundinacea* cultivars, especially Rahela.

The results of many studies confirmed the increase in plant yield under Si fertilization [16,46]. Eneji et al. [47] also reported improvements in the growth and nutrient utilization of four grass species under Si supply and drought conditions. In our study, an increase in the SDM in all the tested species under the influence of the Si applications was observed mainly during the summer drought. 

The average C content in the roots of the tested grasses was low (0.35 g g^−1^) compared to that of the literature (0.45 g g^−1^) (independent of substrate moisture conditions and Si applications) [48]. At the same time, the differences in the RCC values between the grass species were demonstrated. The cultivars of *F. arundinacea* were characterized by the highest carbon content. The obtained results may help to update the data on the C concentration in the plant parts and the C allocation coefficient of plants used in simulation models.

Droughts can lead to an increase in carbon allocation to the roots of grasses and to soils despite varying intensities and durations [49,50,51,52]. However, some studies have also indicated that droughts can reduce the flux of carbon from the grass roots to the soil [53]. Extreme droughts limit the shift of carbon from the aboveground to the belowground composition, but moderate droughts result in carbon being allocated to the organs that are most in need of carbon when under high water stress [54]. Presumably, water deficits decrease carbon sequestration and increase the residence time of newly assimilated carbon in the leaf biomass, thereby increasing the time of carbon partitioning from the plants to the soil [55]. In our study, the grasses exposed to the droughts showed a decrease in carbon partitioning in both the roots and shoots compared to the control. 

According to Li et al. [56] silicon can improve plant growth and enhance plant biomass carbon accumulation under abiotic stress (including droughts). The results suggested that Si-mediated biomass carbon accumulation increases under mild and moderate stress. However, it depends on the plant cultivars, Si dosage, and stress intensity. In our research, the administration of Si under the drought conditions (D + Si) significantly reduced the RCC but stimulated both RCA and SCA, especially in the more stress-sensitive *F. braunii* and *L. perenne* cvs. (Figure 4a,b). 

Prolonged water deficits lead to sugar accumulation and a decrease in the leaf N content. This leads to a C:N imbalance reflected by an increased C:N ratio. The accumulation of soluble sugars and osmolytes during droughts protects plants from structural and functional damage due to dehydration and is considered an osmotic adaptation of plants to water scarcity [57]. Our research also showed a C:N imbalance under the water deficit conditions. Plants exposed to a drought during the first growth phase in the spring (D1) showed a high SNC and a low C:N ratio in their shoots. This was related to the reduction in plant growth and decrease in the TPDM, SDM, and RL. Moreover, Si applications decreased the C:N ratio by 7% in the shoots under stressful conditions, especially in the summer (D2 + Si). The highest values were noted in *F. arundinacea*, both in its shoots and roots.

The Si applications mitigated the negative effects of the droughts in forage grasses by increasing plant growth (SDM, RDM), the allocation of biomass to the roots (RMR), the carbon accumulation in the plant biomass (SCA, RCA), and the water use efficiency (WUE). Simultaneously, in the case of *F. braunii* and *L. perenne*, a significant increase in the length of the roots (RL) and the values of the net photosynthetic rate (*P*_N_) were demonstrated. Moreover, an increase in the root diameter (RD), transpiration rate (*E*), and leaf flavonol content (Flv) were also shown in *F*. *braunii*. Thus, the results indicated that Si supplementation had the strongest effect on the more sensitive cvs. of *F. braunii* and *L. perenne*. It affected the distribution of the plant biomass, the morphometric features, and the physiological parameters.

## 4. Materials and Methods

### 4.1. Experimental Design and Management

The pot experiment was carried out in semi-natural condition in an open foil tunnel located in Warsaw University of Life Sciences (52°09′40″ N, 21°03′14″ E) for two years (2017–2019). Three forage grass species differing in sensitivity to water deficiency were used in the study: tolerant—*Festuca arundinacea* Schreb. (cvs. of Odys and Rahela) [11]—and sensitive—*Lolium perenne* L. (cvs. of Bajka—2n—and Gagat—4n) [10] and *Festulolium braunii* (Richt.) A. Camus (cvs. of Felopa and Sulino) [58]. The seeds were obtained from Danko Plant Breeding Sp. z o.o. z/s in Choryń (cvs. of Odys, Gagat, Felopa, and Sulino) and Grunwald Plant Breeding Sp. z o.o. Grupa IHAR (cvs. of Bajka and Rahela).

The pots (h = 40 and d = 25 cm), with their bases in a closed system, were filled to a weight of 12 kg with a substrate (mixture of sand and soil in the proportion of 1:4, respectively). Luvisol soil with a loamy sand texture [59] was used for the study. The substrate was characterized by a high content of phosphorus, very high magnesium, and low potassium concentration (8.56, 7.45, and 5.81 mg 100 g^−1^ of soil, respectively). It was alkaline (pH = 7.5) with the ratio of C:N = 13. Mineral fertilization with nitrogen, phosphorus, and potassium at dosages equivalent to (kg ha^−1^ per year, respectively) 180, 26.2, and 100 was used in the research. Nitrogen (ammonium nitrate) was applied before each regrowth. Additionally, potassium (potassium chloride) was applied before the first and second regrowth, and phosphorus (triple superphosphate) was applied once before the first regrowth. 

In summer, in the year preceding the measurements, grass seeds were sown. Ten seeds of each individual cultivar were placed in each pot, and, after emergence, the number of seedlings was reduced to 5 (only seedlings of similar growth were left for further research). Plants were cut in October and wintered at approximately 5 °C. The substrate water regime was controlled every day by weighing each pot and adding water as needed to maintain the required level of capillary water capacity (CWC) [60]. In the years of measurements (2018, 2019), the plants were grown under stress-free conditions until May (tillering phase), and then some plants were subjected to droughts and the other were kept under optimal moisture conditions of 70% CWC—control (C). During drought stress, irrigation was withheld, and substrate was allowed to naturally dry. Plants were treated with drought conditions once a year for 21 days: in spring (May) during the first regrowth (D1) or in summer (July) during the second regrowth (D2). In the conditions of each drought, half of the plants were additionally fertilized with silicon (Si) (D1 + Si and D2 + Si) twice: 7 days before drought and during the term when the stress was started. Foliar nutrition with silicon as NanoFYT Si (AGRA GROUP a.s., Střelské Hoštice, Czech Republic) in the form of stabilized nanoparticles of hydrated SiO_2_ (230 g L^−1^) was applied in amounts of 3.5 mL of solution per plant in the dilution recommended by the manufacturer (0.5 L NanoFYT Si 150 L^−1^ H_2_O per ha). After drought, all plants were subjected to well-watered conditions (70% CWC). The plants were cut three times during the growing season in both study years: after the end of D1 and D2 (in mid-May and mid-July, respectively) and also in the last week of September. The tests were carried out under natural light conditions, whereas the air humidity and temperature were recorded with a data logger placed next to the experimental trial (Figure 1).

### 4.2. Methods and Measurements

The chlorophyll (Chl) and flavonol (Flv) content of plants were measured using a Dualex meter (Scientific+, FORCE-A, France) [61]. The results were expressed in Dualex units (values of Chl ranged from 0 to 150, while Flv values ranged from 0 to 3). The portable gas analyzer (LCpro+, ADC BioScientific Ltd., Hoddesdon, UK) was used to determined net photosynthetic rate (*P*_N_; μmol CO_2_ m^−2^ s^−1^), transpiration rate (*E*; mmol H_2_O m^−2^ s^−1^), stomatal conductance (*g*_s_; mol H_2_O m^−2^ s^−1^), and sub-stomatal CO_2_ concentration (*C_i_*; µmol CO_2_ mol air^−1^) [62]. During the measurement, open-gas exchange system was operated in differential mode at a flow rate of 150 mmol s^−1^ of ambient air, and saturating irradiance was about 1000 µmol m^−2^ s^−1^. The parameters were measured after the stabilization of conditions in the chamber. Based on these primary data, water use efficiency (WUE; µmol CO_2_ mmol^−1^ H_2_O) was calculated as a ratio of *P*_N_ to *E*.

Physiological measurements were done on all plants in the pot on the middle part of a young, fully developed leaf always at the same time (8.00–10.00 a.m.) after 4, 7, 14, and 21 days of maintaining the water deficit (4, 7, 14, and 21 days after treatment—DAT). 

Plants from pots were cut (at 5 cm above the soil surface) in each regrowth after a period of water deficiency and after third regrowth. The harvested biomass was then dried (105 °C for 72 h) and weighed to determine the shoot dry mass (SDM; g plant^−1^). Relative water content (RWC; %) was estimated on the basis of fresh, turgid (after submerging leaves for 12 h in distilled water at room temperature (20 °C)), and dry masses of plant according to the following equation [63] (Equation (1)):

(1) $$\mathrm{RWC}\;=\frac{\mathrm{leaf}\;\mathrm{fresh}\;\mathrm{mass}\;-\;\mathrm{leaf}\;\mathrm{dry}\;\mathrm{mass}}{\left(\mathrm{turgid}\;\mathrm{mass}\;-\;\mathrm{leaf}\;\mathrm{dry}\;\mathrm{mass}\right)}\times 100$$

Root samples were taken after the last (third) regrowth once a year in October. Plant roots were separated from the soil by washing them on sieves (mesh diameter of 3–0.3 mm) in stream of water and were manually cleaned of organic parts. The fresh roots were then scanned with an optical scanner and analyzed using the WinRHIZO 2012 (Regent Instruments Inc., Canada) software. Total length (RL; m) and average diameter (RD; mm) of the roots were determined. Roots of grass cultivars were sorted into ten size classes according to their diameter (0.0 <= 0.3, 0.3 <= 0.6, 0.6 <= 0.9, 0.9 <= 1.2, 1.2 <= 1.5, 1.5 <= 1.8, 1.8 <= 2.1, 2.1 <= 2.4, 2.4 <= 2.7, and 2.7 <= 3 mm) to estimate root distribution [6]. Then, the roots were dried at 105 °C for 72 h, and root mass (RDM; g plant^−1^) was estimated. The specific root length (SRL; m g^−1^) was calculated as the ratio of RL to RDM, while root mass ratio (RMR; g g^−1^), expressing the relative amount of biomass allocated to the belowground organs, was calculated as the ratio of RDM to total plant dry mass (TPDM).

The content of carbon (C; g kg^−1^ DM) and nitrogen (N; g kg^−1^ DM) in roots and shoots of plants were measured using the CNS elemental analyzer (Vario MACRO CNS, Hanau, Germany). Based on these data the C:N ratio was determined. The carbon accumulation (kg plant^−1^) in the roots (RCA) and shoots (SCA) of plants was evaluated based on the carbon content and dry mass of particular organs. Measurements were made once a year after the third regrowth. 

### 4.3. Statistical Analysis

Experiment was established in a completely randomized design with three replications (90 pots per year). The data presented are the mean values from the two years of investigations as a result of a similar reaction of the plants examined with the applied water and Si conditions. Values obtained concerned 23 features analyzed in 6 cultivars within 3 grass species and 5 variants of water and Si conditions. Multifactor analysis of variance (ANOVA) was performed using the TIBCO Statistica™ 13.3.0 software. Tukey’s multiple comparison test was used to compare the mean values (*p* ≤ 0.05). A principal component analysis (PCA) was performed to evaluate the relationships between all the tested parameters and to characterize the multivariate differences between them and conditions of drought and silicon applications.

## 5. Conclusions

The improvement in some of the physiological and growth characteristics of *F. braunii*, *F. arundinacea*, and *L. perenne* after the foliar application of Si confirmed its suitability for usage in water-stressed environments. Silicon’s positive effect was linked to a reduction in oxidative stress due to the limitation of excessive water loss through transpiration. Our findings implied that increasing the root length and biomass may help forage grasses function better under drought stress. These findings also implied that the Si applications allowed greater C assimilation which was then preferentially transferred to the roots to develop the fine root network.

Si could help grasses adapt to droughts by enhancing the allocation of carbon to the roots and increasing the water usage efficiency. However, more research into the effects of Si on the development and productivity of additional forage grass species under varying soil water conditions is required.

## Figures and Tables

**Table 1:** Mean values of the studied features according to water (C—control; D1 and D2—water deficits) and silicon (Si) conditions, grass species (*F. braunii*—*F.b*; *F. arundinacea*—*F.a*; *L. perenne—L.p*), and cultivars (Felopa, Sulino, Odys, Rahela, Bajka, Gagat) regardless of the others.

Features	Water and Si Conditions					Species			Cultivars					
	C	D1	D1 + Si	D2	D2 + Si	*F.b*	*F.a*	*L.p*	Felopa	Sulino	Odys	Rahela	Bajka	Gagat
Chl	23.2 ^c^	21.4 ^b^	20.4 ^b^	12.1 ^a^	12.0 ^a^	18.8 ^b^	21.4 ^c^	15.9 ^a^	18.7 ^b^	18.9 ^bc^	20.9 ^cd^	21.8 ^d^	14.8 ^a^	17.0 ^b^
Flv	1.75 ^c^	1.11 ^a^	1.12 ^a^	1.28 ^b^	1.22 ^ab^	1.35 ^b^	1.68 ^c^	1.07 ^a^	1.33 ^b^	1.38 ^b^	1.67 ^c^	1.70 ^c^	1.03 ^a^	1.11 ^a^
*P*_N_	8.34 ^b^	2.53 ^a^	2.70 ^a^	1.92 ^a^	2.34 ^a^	3.28 ^a^	4.67 ^b^	5.12 ^b^	3.32 ^a^	3.24 ^a^	4.58 ^ab^	4.76 ^ab^	5.29 ^b^	4.95 ^b^
*E*	2.40 ^d^	1.63 ^c^	1.32 ^b^	0.74 ^a^	0.90 ^a^	1.13 ^a^	1.77 ^b^	1.79 ^b^	1.06 ^a^	1.19 ^a^	1.72 ^b^	1.81 ^b^	1.94 ^b^	1.65 ^b^
*C_i_*	512 ^a^	524 ^ab^	518 ^a^	552 ^c^	541 ^bc^	405 ^a^	460 ^b^	714 ^c^	410 ^a^	400 ^a^	462 ^b^	457 ^b^	791 ^d^	637 ^c^
*g*_s_	0.217 ^c^	0.083 ^b^	0.075 ^ab^	0.039 ^a^	0.042 ^a^	0.100 ^a^	0.121 ^a^	0.115 ^a^	0.096 ^a^	0.104 ^a^	0.123 ^a^	0.119 ^a^	0.123 ^a^	0.108 ^a^
WUE	3.75 ^d^	1.35 ^a^	2.07 ^b^	2.58 ^c^	3.39 ^d^	2.82 ^a^	2.67 ^a^	2.93 ^a^	3.09 ^b^	2.54 ^a^	2.66 ^ab^	2.68 ^ab^	2.81 ^ab^	3.05 ^b^
RWC	81.2 ^d^	25.0 ^b^	27.3 ^c^	15.5 ^a^	16.1 ^a^	41.9 ^b^	39.3 ^a^	40.8 ^b^	42.2 ^c^	41.7 ^bc^	39.4 ^a^	39.2 ^a^	40.1 ^ab^	41.5 ^bc^
RD	0.447 ^ab^	0.432 ^a^	0.452 ^b^	0.452 ^b^	0.452 ^b^	0.446 ^b^	0.464 ^c^	0.431 ^a^	0.448 ^bc^	0.444 ^abc^	0.472 ^d^	0.456 ^cd^	0.436 ^ab^	0.426 ^a^
RL	559 ^e^	278 ^a^	320 ^b^	388 ^c^	448 ^d^	384 ^b^	311 ^a^	500 ^c^	349 ^a^	420 ^b^	305 ^a^	317 ^a^	518 ^c^	481 ^c^
RDM	9.47 ^e^	3.65 ^a^	5.14 ^b^	5.64 ^c^	6.80 ^d^	5.55 ^a^	7.11 ^b^	5.74 ^a^	5.32 ^a^	5.78 ^b^	7.36 ^e^	6.86 ^d^	5.24 ^a^	6.25 ^c^
SDM	8.42 ^d^	3.54 ^a^	3.40 ^a^	5.85 ^b^	6.71 ^c^	5.76 ^b^	5.75 ^b^	5.24 ^a^	5.97 ^c^	5.56 ^b^	5.37 ^b^	6.13 ^c^	5.02 ^a^	5.47 ^b^
TPDM	21.6 ^e^	9.1 ^a^	10.8 ^b^	14.5 ^c^	16.9 ^d^	14.1 ^a^	15.6 ^b^	14.0 ^a^	14.0 ^ab^	14.2 ^b^	15.6 ^c^	15.7 ^c^	13.3 ^a^	14.6 ^b^
SRL	62.6 ^a^	78.6 ^c^	64.9 ^a^	71.6 ^b^	67.0 ^ab^	70.7 ^b^	45.7 ^a^	90.3 ^c^	68.5 ^b^	72.8 ^b^	43.3 ^a^	48.2 ^a^	100.3 ^d^	80.3 ^c^
RMR	0.435 ^b^	0.398 ^a^	0.472 ^c^	0.396 ^a^	0.403 ^ab^	0.395 ^a^	0.453 ^b^	0.408 ^a^	0.382 ^a^	0.408 ^b^	0.468 ^d^	0.437 ^c^	0.396 ^ab^	0.420 ^bc^
RCC	383 ^d^	371 ^cd^	352 ^bc^	347 ^b^	319 ^a^	348 ^a^	364 ^b^	348 ^a^	347 ^ab^	348 ^ab^	365 ^c^	364 ^c^	359 ^bc^	337 ^a^
RNC	12.3 ^a^	15.9 ^c^	15.2 ^c^	15.1 ^c^	13.4 ^b^	15.5 ^c^	13.3 ^a^	14.3 ^b^	15.8 ^d^	15.2 ^cd^	13.0 ^a^	13.7 ^ab^	14.5 ^bc^	14.0 ^ab^
C:N root	31.4 ^b^	23.6 ^a^	23.4 ^a^	23.3 ^a^	23.9 ^a^	22.8 ^a^	27.7 ^c^	24.7 ^b^	22.3 ^a^	23.3 ^ab^	28.7 ^e^	26.8 ^d^	25.1 ^cd^	24.3 ^bc^
SCC	406 ^a^	409 ^a^	408 ^a^	409 ^a^	406 ^a^	407 ^a^	408 ^a^	408 ^a^	406 ^a^	408 ^a^	410 ^a^	405 ^a^	411 ^a^	406 ^a^
SNC	18.0 ^b^	29.2 ^d^	26.3 ^c^	14.1 ^a^	15.0 ^a^	21.7 ^b^	18.3 ^a^	21.4 ^b^	21.6 ^b^	21.8 ^b^	18.6 ^a^	18.0 ^a^	21.9 ^b^	20.9 ^b^
C:N shoot	22.6 ^b^	14.5 ^a^	15.8 ^a^	29.5 ^d^	27.4 ^c^	21.1 ^a^	23.6 ^b^	21.0 ^a^	21.4 ^a^	20.8 ^a^	23.7 ^b^	23.5 ^b^	20.7 ^a^	21.4 ^a^
RCA	3.66 ^d^	1.35 ^a^	1.82 ^b^	1.94 ^b^	2.16 ^c^	1.93 ^a^	2.62 ^b^	2.02 ^a^	1.85 ^a^	2.01 ^ab^	2.73 ^d^	2.51 ^c^	1.88 ^a^	2.15 ^b^
SCA	3.42 ^d^	1.45 ^a^	1.39 ^a^	2.39 ^b^	2.72 ^c^	2.34 ^b^	2.35 ^b^	2.14 ^a^	2.42 ^c^	2.26 ^b^	2.20 ^ab^	2.49 ^c^	2.06 ^a^	2.21 ^b^

The means marked with the same lower-case letters (within water and Si conditions, species, or cultivars) did not differ significantly (*p* ≤ 0.05). Chl—content of chlorophyll (Dualex unit), *C*_i_—sub-stomatal CO_2_ concentration (µmol CO_2_ mol^−1^ air), *E*—transpiration rate (mmol H_2_O m^−2^ s^−1^), Flv—content of flavonols (Dualex unit), *g*_s_—stomatal conductance (mol H_2_O m^−2^ s^−1^), *P*_N_—net photosynthetic rate (µmol CO_2_ m^−2^ s^−1^), RCA—root carbon accumulation (kg plant^−1^), RCC—root carbon content (g kg^−1^ DM), RD—root diameter (mm), RDM—root dry mass (g plant^−1^), RL—total root length (m plant^−1^), RMR—root mass ratio (g g^−1^), RNC—root nitrogen content (g kg^−1^ DM), root C:N—root carbon-to-nitrogen ratio, RWC—relative water content (%), SCA—shoot carbon accumulation (kg plant^−1^), SCC—shoot carbon content (g kg^−1^ DM), SDM—shoot dry mass (g plant^−1^), shoot C:N—shoot carbon-to-nitrogen ratio, SNC—shoot nitrogen content (g kg^−1^ DM), SRL—specific root length (m g^−1^), TPDM—total plant dry mass (g plant^−1^), and WUE—water use efficiency (µmol CO_2_ mmol^−1^ H_2_O).

**Table 2:** Significance of the effect of factors and their interactions on the analyzed variables based on the analysis of variance.

Factors	Variable																						
	Chl	Flv	*P*_N_	*E*	*C_i_*	*g*_s_	WUE	RWC	RD	RL	RDM	SDM	TPDM	SRL	RMR	RCC	RNC	C:N root	SCC	SNC	C:N shoot	RCA	SCA
Species (A)	*	*	*	*	*	ns	ns	*	*	*	*	*	*	*	*	*	*	*	ns	*	*	*	*
Cultivar (B)	*	*	*	*	*	ns	*	*	*	*	*	*	*	*	*	*	*	*	ns	*	*	*	*
Condition (C)	*	*	*	*	*	*	*	*	*	*	*	*	*	*	*	*	*	*	ns	*	*	*	*
A × C	*	*	*	*	*	*	*	*	*	*	*	*	*	*	*	*	*	*	ns	ns	ns	*	*
B × C	ns	ns	ns	ns	ns	ns	ns	*	*	*	*	*	*	*	*	*	*	*	ns	*	*	*	*

*—significant effect (*p* ≤ 0.05); ns—not significant; Chl—content of chlorophyll, *C*_i_—sub-stomatal CO_2_ concentration, *E*—transpiration rate, Flv—content of flavonols, *g*_s_—stomatal conductance, *P*_N_—net photosynthetic rate, RCA—root carbon accumulation, RCC—root carbon content, RD—root diameter, RDM—root dry mass, RL—total root length, RMR—root mass ratio, RNC—root nitrogen content, root C:N—root carbon-to-nitrogen ratio, RWC—relative water content, SCA—shoot carbon accumulation, SCC—shoot carbon content, SDM—shoot dry mass, shoot C:N—shoot carbon-to-nitrogen ratio, SNC—shoot nitrogen content, SRL—specific root length, TPDM—total plant dry mass, and WUE—water use efficiency.

## Data Availability

Not applicable.
